# Open peer review urgently requires evidence: A call to action

**DOI:** 10.1371/journal.pbio.3002255

**Published:** 2023-10-04

**Authors:** Tony Ross-Hellauer, Lex M. Bouter, Serge P. J. M. Horbach

**Affiliations:** 1 Graz University of Technology, Graz, Austria; 2 Know-Center GmbH, Graz, Austria; 3 Amsterdam University Medical Center, Amsterdam, the Netherlands; 4 Vrije Universiteit, Amsterdam, the Netherlands; 5 Aarhus University, Aarhus, Denmark

## Abstract

Open Peer Review is gaining prominence in attention and use, but to responsibly open up peer review there is an urgent need for additional evidence. This Perspective proposes a preliminary research agenda and issues a call-to-action.

## Teaser

Open Peer Review is gaining prominence in attention and use, but to responsibly open up peer review, there is an urgent need for additional evidence. Here, we propose a preliminary research agenda and issue a call to action.

This article is part of the *PLOS Biology* 20th Anniversary Collection.

Open Science innovations such as open methods, code, and data aim to reduce questionable and problematic research practices and build trustworthiness through increased transparency and accountability of research [1]. Scholarly journals have an important role in facilitating and using Open Science practices. Peer review is their key tool in this, despite the known deficiencies of what ultimately remains a subjective system of appraisal by a small number of invited experts. To improve peer review in accordance with the open science agenda, various forms of Open Peer Review (OPR) have been proposed. While having the potential to positively contribute to the trustworthiness and validity of publications, the efficacy of OPR in this regard is still largely unknown. In this Perspective, we therefore argue for the urgent need for additional evidence on OPR in order to open up peer review responsibly.

While we tend to think of peer review as a constant in scholarly life, models of review have been in flux since their inception in an effort to accommodate diverse and changing expectations [2]. Despite the position of peer review as the “gold standard” of scholarly quality assurance, the various models used remain far from optimal. They suffer from issues ranging from slowness, bias, inconsistency, and suboptimal quality to a lack of interest, time, expertise, and reward.

A main limitation to optimizing peer review practices is a fundamental lack of knowledge [3]. For a start, there is still no shared understanding of what constitutes good review quality. Similarly, debates on the expertise needed to perform a high-quality review, or the social, epistemic, or personal factors hindering the production of “good” reviews, are far from settled [4]. These, and other limitations, imply that advocates or opponents of reform in peer review often do not appreciate the paucity of available evidence and rely on generalizations or ill-founded intuitions [3]. This also holds true for the risks and benefits of the three main models of OPR: Open Identities, in which the identities of actors involved are disclosed; Open Reports, in which review reports are publicly made available; and Open Participation, in which noninvited or nontraditional wider audiences are involved [5].

OPR has seen a rise in interest in recent years [6] and has particularly gained prominence within journals but is also used for reviewing books and conference abstracts. In general, OPR models aim at increasing transparency or participation in review processes. All should be considered distinct innovations with their own potential advantages and drawbacks that can be combined in multiple ways. Whereas revealing identities aims primarily at increasing accountability and mitigating conflicts of interest, publishing review reports aims at greater transparency of review processes for readers. Open participation aims to avoid closed, siloed review communities and bring in a broader range of perspectives [5]. OPR models are also increasingly the de facto standard for related innovations, including preprint peer review, registered reports [7], and postpublication peer review [8]. In addition, although less standard in practice, recent work indicates an interest in applying these principles to peer review of data, code, and funding applications [9].

Despite these high expectations, the evidence-base on which such claims rest is very thin [6,10]. In particular, there is still minimal information available on the fundamental question of the extent to which OPR affects the quality and trustworthiness of the items under review.

Additional areas of concern exist for each type of OPR. For Open Identities, the question of whether and how they might increase status bias in review processes will be important to answer. The ubiquitous concern that more junior reviewers will either limit their criticisms of more established scholars or risk reprisals from the latter at a later point in their careers has barely been investigated. There is, however, evidence that, given the option to sign, senior or male authors are more likely to do so. Hence, there is an urgent need to further examine how power dynamics affect review processes under conditions of Open Identities. For Open Reports, whether and how they might improve publication quality and trustworthiness remains an open question. Equally important will be how they function to build trust among broader reading publics. For Open Participation, evidence is very scant. Hence, there is a need to investigate how such crowdsourcing can be successfully scaled, whether it leads to more diversity, and how these processes affect the quality and trustworthiness of the items under review.

Failure to perform empirical research on these questions will likely lead to implementation in the absence of evidence. Innovation is essential, and OPR has the potential to bring much good but may also have undesirable side effects. Without sound evidence, we risk not only wasting resources but could also potentially worsen the situation.

As the arc of scholarly communication bends towards greater transparency and openness, addressing these evidence gaps is urgently necessary. We call upon all stakeholders—particularly researchers, publishers, and funders—to contribute in achieving this aim. To inspire that process, we have sketched out a preliminary research agenda containing what we assess to be the most pressing questions, including the most promising research approaches to be used in addressing them. The initial step should be to extend and validate the agenda itself through cocreative methods. Although our prioritization of questions and methods draws heavily from a recent preprint that surveys the literature [10], we believe that to maximize uptake, the plan of action should be community driven.

Before executing the research agenda, we should collaboratively agree on core outcomes. Such a core outcome set should not be designed too narrowly, so as not to compromise the diversity of modes of knowledge production and the inherent quality criteria that they encompass. Yet, a certain degree of standardization would facilitate subsequent systematic reviews and meta-analyses of studies testing the effects of transparency and other interventions designed to improve the effectiveness of peer review. Examples of core outcomes range from the quality of the review reports, the impact on the item under review, and the detection of problematic research practices to process parameters such as acceptance rates of review invitations, time taken to perform the review, or satisfaction of reviewers, editors, and authors. Following this preliminary work, the community should work together to study concerns related to OPR (Box 1).

**Box 1. Key priorities for research on Open Peer Review**

**How do variants of OPR affect the content and quality of the review process?**

Addressing this question requires either large-scale observational studies or, when feasible, randomized controlled trials across multiple epistemic communities, publishing contexts, and stakeholders. Because these studies are both resource intensive and dependent on the availability of sufficient data, we propose both funding agencies and scholarly communication stakeholders (e.g., publishers, preprint servers, and peer review platforms) to collectively engage in this endeavor.

**What are the implications of OPR elements for reviewers, authors, and other stakeholders involved in the review process?**

Due to a lack of evidence either way, no definitive conclusions can yet be drawn regarding the existence or consequences of backbiting or blunted criticisms, e.g., in models with Open Identities. As such, fears have a major role in perceptions of OPR; finding out if and how they are substantiated by evidence should be a priority. In addition, the effect of OPR elements on power imbalances requires in-depth analysis, as demographics do seem to have a heavy role in who opts to engage with OPR models.

**How do models of OPR apply to review of objects other than traditional journal articles?**

This includes preprint peer review and publish–review–curate models but also review of objects such as datasets, software/code, and monographs, as well as review processes in funding and hiring contexts. As many of these contexts and models are likely to gain prominence in the future, and some of them (e.g., preprint peer review) use certain elements of OPR more or less by default, a better understanding of the merits and consequences of OPR in these settings is urgently needed.

OPR, understood as the application of Open Science principles to increase transparency and participation in peer review, seems to have rich potential to help in reshaping scholarly communication towards greater trustworthiness and validity. Yet, despite increased uptake of OPR models, scholarly work to assess their effectiveness has not kept pace. We hope our provisional research agenda may inspire collaborative efforts to address this.
